# A Preliminary Pharmacokinetic Study of Betulin, the Main Pentacyclic Triterpene from Extract of Outer Bark of Birch (*Betulae alba* cortex) 

**DOI:** 10.3390/molecules13123224

**Published:** 2008-12-18

**Authors:** Sebastian Jäger, Melanie N. Laszczyk, Armin Scheffler

**Affiliations:** 1 Carl Gustav Carus-Institut, Am Eichhof 30, D-75223 Niefern-Öschlbronn, Germany; E-mail: info@carus-institut.de (A. S.); 2 Betulin-Institut, Blumenstrasse 24, D-64297 Darmstadt, Germany; E-mail: m.laszczyk@betulin-institut.de (M-N. L.)

**Keywords:** Triterpene extract, Betulin, Preliminary pharmacokinetics, Subchronic toxicity

## Abstract

During the last two decades triterpenes have attracted attention because of their pharmacological potential. Triterpene extract (TE) from outer bark of birch consisting mainly of betulin is able to form an oleogel which was successfully tested in the treatment of actinic keratosis. Some aspects of TE *in vitro* pharmacology are already known. Now we show preliminary pharmacokinetics of betulin and results of a subchronic toxicity study of TE in rats and dogs. Because of poor aqueous solubility of the TE-triterpenes (< 0.1 µg/mL respectively), for pharmacokinetic studies it was suspended in sesame oil (rats, i.p.) and PEG 400 / 0.9 % NaCl (dogs, s.c.). I.p. administered, betulin, the main component of TE, shows time dependency over a period of 4 h and reaches a dose-independent serum level of 0.13 µg/mL. Dose dependency was observed with s.c. administration. At 300 mg/kg a maximum plasma concentration of 0.33 µg/mL betulin was detected after 28 daily applications. The subchronic toxicity study showed no toxicity of TE in rats (i.p.) and dogs (s.c.). In conclusion, triterpene extract from birch bark is safe, its betulin is bioavailable and in addition to published triterpene biological activities TE provides high potential for further pharmaceutical and pharmacological research.

## Introduction

Outer bark of birch (*Betula alba* cortex) contains pentacyclic triterpenes, mainly betulin (BE, up to 34 %), but also betulinic acid (BA), oleanolic acid (OA), lupeol (LU) and erythrodiol (ER). They can be extracted as a triterpene rich dry extract (TE) which is able to form a topically applicable oleogel [1,2]. The birch triterpenes have known antiviral, antimicrobial and hepatoprotective pharmacological activities [3,4,5]. BA, OA or BE also have antitumor effects [2,6,7]. These triterpenes show anti-inflammatory activities, as do others such as ER or LU [7,8,9,10]. Such properties are of interest in treating skin diseases where a topical application is preferred. Therefore the bioavailability and toxicity of the birch triterpene extract is of interest. The toxicity of triterpenes is reportedly relatively low. A 600 mg/kg i.p. dose is well tolerated [11,12,13]. The LD_50_ for OA is 1,500 mg/kg (i.p.; mouse) [14]. Only a few pharmacokinetic studies have been published. BA was found in various tissues 24 h after i.p. administration (500 mg/kg; mouse) and reached its highest concentration in perirenal fat. Peak serum concentration (4.0 µg/mL) was observed at 0.23 h after application [15]. These findings indicate that triterpenes are of relatively low toxicity, and therefore can generally be used therapeutically. However, their solubility is low why their bioavailability is questionable. The solubility in water of OA and BA is only 0.02 µg/mL [16]. In animal experiments triterpenes are administered i.p. or s.c. as a dispersion, whereas i.v. they have to be dissolved. Therefore, organic solvents, for example *N*,*N-*dimethyl-acetamide, are necessary [17], but they are not excipients of first choice for pharmaceutical usage [18]. In contrast, the solubility of BE in oil is approximately 3 mg/mL [2] which offers the possibility of topical application. The clinical relevance of dermally applied triterpene extract of the outer bark of birch has been shown previously in treating actinic keratosis [19,20]. A first step to learn more about its way of action is the examination of the bioavailability in animal models. Furthermore animal models provide the possibility to study toxicity. Therefore TE was investigated in a subchronic toxicity study which was combined with a preliminary pharmacokinetic study of its main component BE in rats (i.p.) and dogs (s.c.). These data are presented here.

## Results and Discussion

### Dry triterpene extract

**Figure 1:**
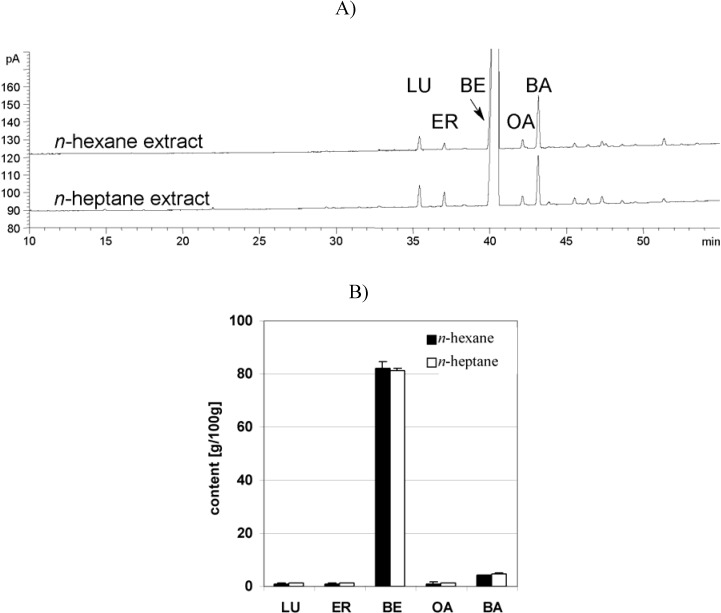
Qualitative and quantitative comparison of triterpene distribution in TE depending on extraction solvent (GC-FID). A) Chromatogram of *n-*hexane- and *n-*heptane extract. B) Quantitative comparison of *n-*hexane- and *n‑*heptane extract. Standard deviation is displayed as error bars of *n-*hexane (4 batches) and *n-*heptane extracts (6 batches); error bars: SD.

The pharmacokinetic studies presented here were performed with TE extracted with *n-*hexane, whereas the TE used in the other publications with *n-*heptane [2,19,20]. Therefore it was necessary to determine whether the two solvents give the same results. Four *n-*hexane extracts were compared with 6 *n-*heptane extracts as shown in Figure 1. The values of LU, ER, BE, OA and BA are equal, showing that the extraction of TE is independent of the extraction solvent by t-test (p = LU 0.19; ER 0.38; BE 0.55; OA 0.75; BA 0.06).

**Figure 2:**
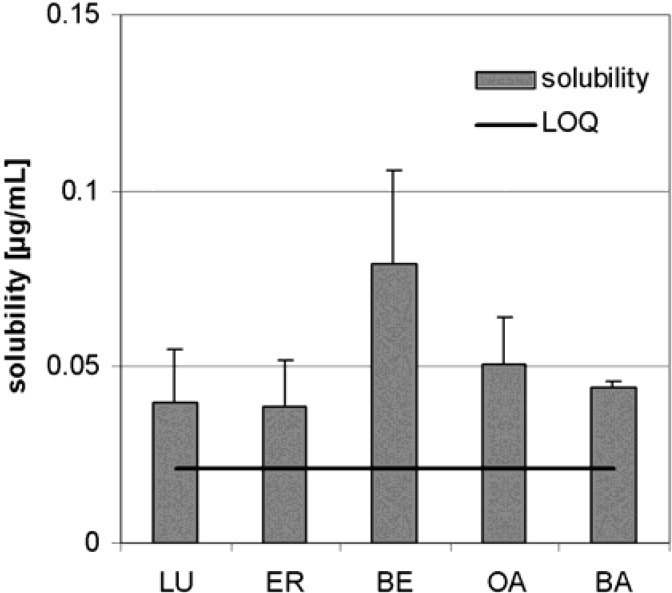
Aqueous solubility of TE (n = 3, error bars: SD).

The solubility of TE in water was previously unknown, but it is important for development of formulations and for the estimation of its pharmacokinetics. In distilled water, triterpenoids from TE were soluble as shown in Figure 2. BE is soluble to only 0.08 µg/mL (n = 3). The added amount of TE was sufficient because no triterpene reached the concentration that would otherwise be expected from the amount added.

### Preliminary pharmacokinetics in rats

**Figure 3:**
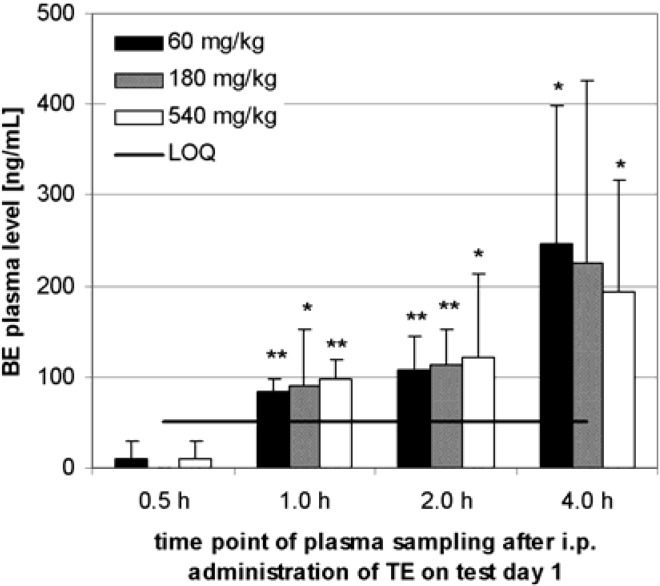
Plasma BE levels after i.p. administration to 6 Sprague-Dawley rats (3 male, 3 female) in each dose level group. Samples were taken at the specified time after administration on test day 1. Error bars: CI; The increase of BE plasma level in comparison to the level after 0.5 h is shown by t-test.

TE was administered for 28 days to rats (i.p.) for the determination of plasma BE levels in three dose level groups. Each dose level group contained three male and three female animals. The BE plasma level showed no dependency on the sex of the animals thus it was not examined further. In rats there was a time dependency of the BE plasma level in the range of 0.5 h to 4 h (see Figure 3). Within 1, 2 or 4 h on test day 1, a significant or highly significant increase of BE concentration in comparison to the value after 0.5 h was observed in all three dose level groups.

**Figure 4:**
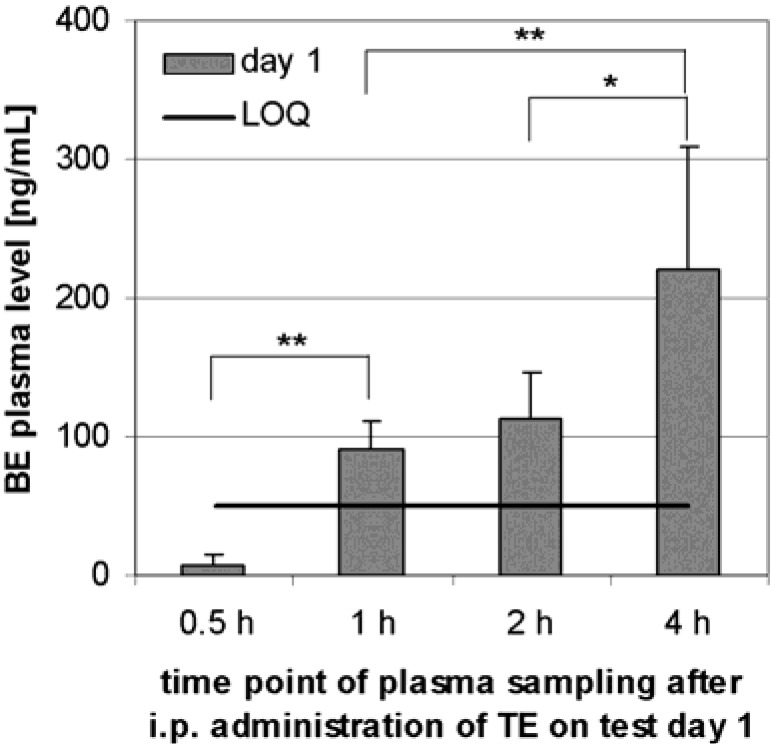
Plasma BE levels after i.p. administration to 18 Sprague-Dawley rats (9 males, 9 females). Samples were taken at the specified time after administration on test day 1. Error bars: CI.

No dose dependency was observed. Therefore the average BE concentration was calculated over all dose level groups as shown in Figure 4. The average plasma BE level rose significantly within the first 4 h after application to 221 ng/mL (CI: 89 ng/mL, n = 18) (see Figure 4).

**Figure 5:**
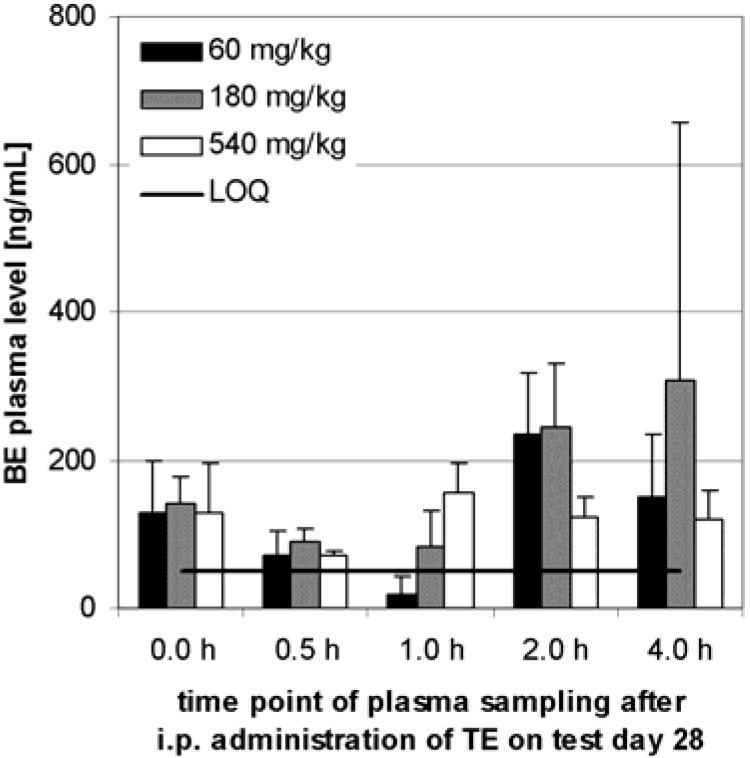
Plasma BE levels after i.p. administration to 6 Sprague-Dawley rats (3 males, 3 females) in each dose level group. Samples were taken before the 28th day administration and at the specified time after administration on test day 28. Error bars: CI.

Before the twenty-eighth application, in all dose level groups, average concentrations between 129 ng/mL and 141 ng/mL were obtained, but there were no significant differences (Figure 5). Within 4 h after the twenty-eighth application no time and dose dependent increase of BE plasma level was observed. At the end of the test, no significant difference of BE level was detected in comparison to the value before injection on that day.

### Preliminary pharmacokinetics in dogs

TE was administered s.c. daily for 28 days to dogs. Each dose level group contained three male and three female animals. The BE plasma level showed no dependency on the sex of the animals thus it was not examined further. No BE was found in the plasma before administration whereas some animals developed a BE-plasma level above the LOQ (50 ng/mL) after the first administration.

**Figure 6:**
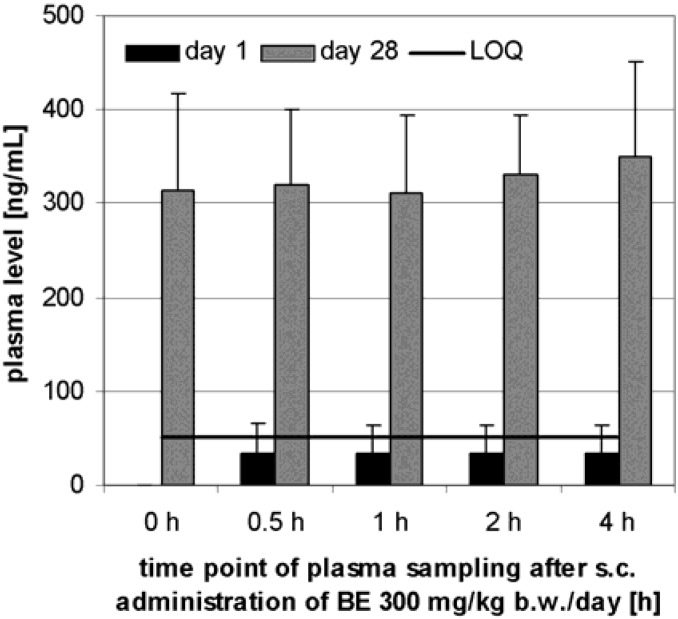
Plasma BE levels after s.c. administration of 300 mg/kg TE to 6 Beagle dogs (3 males, 3 females). Samples were taken before administration and at the specified time after administration on test day 1 and 28. Error bars: CI.

The plasma concentration in all dose level groups did not change in the period between 30 min and 4 h after administration as shown in Figure 6 for the highest dose level group chosen as an example. Therefore the results at different times after administration on day 1 and 28 were pooled for the determination of dose dependency.

**Figure 7:**
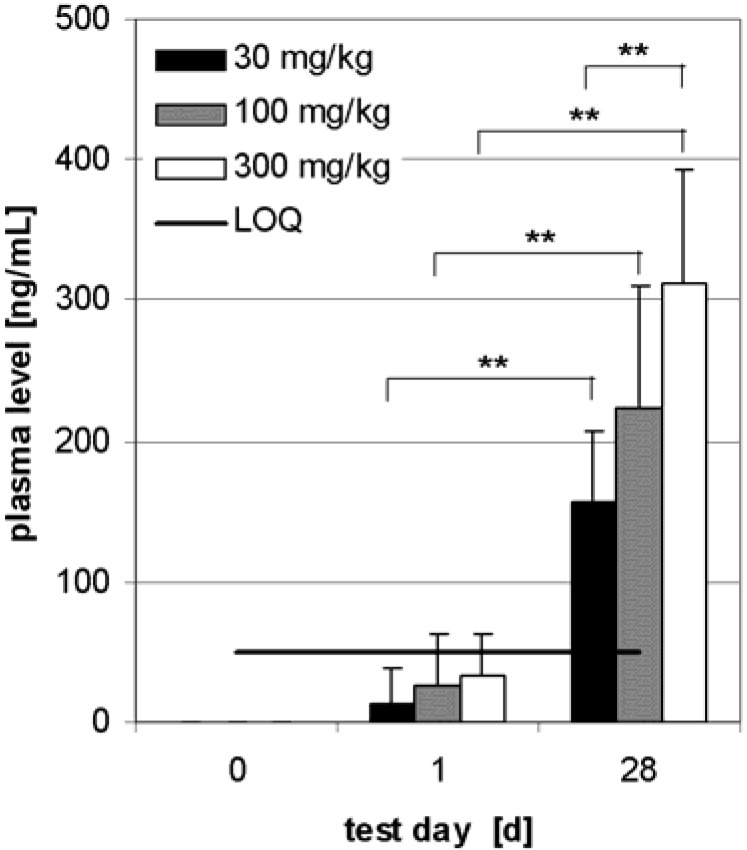
Plasma BE levels before application, on day 1 and day 28 after s.c. administration of TE to 6 Beagle dogs (3 males, 3 females) in each dose level group. The dose level groups were 30, 100 and 300 mg/kg and plasma sampling was carried out within the first 4 h after administration. Error bars: CI.

Unlike with i.p. administration, an increasing BE level was observed depending on the number of s.c. applications in each dose level group (Figure 7). Also a highly significant dose dependency (30 ‑ 300 mg/kg) of plasma BE concentration was shown after 28 days. On day 28, plasma concentration reached 165 ± 43 ng/mL BE in dose level group 30 mg/kg, 234 ± 93 ng/mL BE in dose level group 100 mg/kg and 325 ± 82 ng/mL BE (range: CI, n = 6 respectively) in dose level group 300 mg/kg.

### Subchronic toxicity studies

With rats, daily i.p. doses of TE up to 540 mg/kg for 28 days produced no toxic symptoms or mortality and no histopathological changes were seen. At all doses, an inflammatory reaction was observed in the abdominal cavity due to local irritations by unsolved triterpene particles (see Table 1). The macroscopic post-mortem examination revealed a whitish-yellow oily aqueous liquid in the abdominal cavity in the animals of the control group and all BE treated groups. In addition, disperse areas with whitish deposits and discolourations and adhesions in various abdominal organs were observed in all BE treated groups. These findings are considered as effects of the vehicle and/or test substance deposits. A dose related increase was noted in the absolute and relative weights of spleen. No test substance-related findings were noted at histopathological examination. Inflammation in the abdominal cavity of all rats of the high dose group correlated with the results of macroscopic post-mortem examinations.

**Table 1:** Examinations after 28 days of daily TE administration within subchronic toxicity studies in comparison to the parallel administration of vehicle.

parameter	intraperitoneal (i.p.) administration to Sprague-Dawley rats			subcutananeous (s.c.) administration to beagle dogs		
dose level group	60 g/kg	180 g/kg	540 g/kg	30 g/kg	100 g/kg	300 g/kg
leucocytes	n.c.	+ 46 % ♀ n.c. ♂	+ 45 % ♀ + 53 % ♂	n.c.	n.c.	n.c. ♀ + 369 % ♂
platelet count	n.c.	+ 31 % ♀ + 34 % ♂	+ 30 % ♀ + 45 % ♂	n.c.	n.c.	n.c.
Differential blood count	n.c.	n.c.	n.c.	n.c.	n.c.	n.c.

n.c.: no significant change; ♀: female animals; ♂: male animals

With beagle dogs, daily dose levels up to 300 mg/kg were tolerated and there were also no histopathological changes. Haematological and clinical-chemical parameters showed a dose-dependent inflammatory reaction due to triterpene particles (see Table 1). TE dispersion in PEG 400 (0.9 % NaCl) produced pronounced inflammatory reactions at the application sites. The body weight was not significantly reduced. The macroscopic post-mortem examination revealed wheals at the injection sites in all animals which is a result of the insolubility of the test substance. The histopathology revealed no systemic test substance-related morphological changes in any of the dogs, but the subcutis of the skin at injection sites revealed a pronounced inflammatory reaction due to the irritating potential of the triterpenes of TE, which were not totally absorbed. In addition to subchronic toxicity, acute and subacute toxicity studies as well as skin sensitization (Magnusson and Kligman), mutagenesis studies (Ames test, in vitro human lymphocyte test, in vivo micronucleus test) were performed as required for clinical investigations. TE showed no effect in all pharmacological safety studies at concentrations up to 540 mg/kg (i.p.) and 300 mg/kg (s.c.), respectively.

## Discussion

Birch bark triterpenes were prepared as a dry triterpene extract (TE) by solvent extraction. It was possible to compare the data obtained for the pharmacokinetics of TE extracted with *n-*hexane with the published biological effects, even though in the latter case the extract was obtained by *n-*heptane, because no significant solvent-dependent difference in their composition could be found. Surprisingly, there are no significant (p ≥ 0.10) differences in the aqueous solubilities of TE triterpenes depending on their molecular structures. The recently published solubility of OA and BA was determined as 0.02 µg/mL [16], thus showing a comparable dimension to the results presented here.

Despite its solubility of less than 0.10 µg/mL, a BE serum level between 0.13 and 0.14 µg/mL was obtained by i.p. administration after 28 days to rats. S.c. administration even obtained a concentration of 0.33 µg/mL in dogs. It is known that OA is able to bind to plasmin and albumin [17,21], possibly explaining such high serum concentrations. Because of different absorption mechanisms by s.c. and i.p. administration, and the application to rats and dogs, the observed differences in serum concentrations are not surprising.

Within 4 h after i.p. administration to rats, a time dependent serum level developed. Neither dose dependence nor dependence on numbers of applications could be detected. It seems to reach a saturation of 138 ng/mL (CI: 29 ng/mL, n = 90) BE in plasma. The plasma level may be maintained by peritoneal absorption into the serum and accumulation in different tissues [15], or possibly by metabolism [11]. Our data on BE in rats and dogs do not accord with published data on the single substance BA in mice, where serum levels of 2 to 4 µg/mL were shown after i.p. administration [15,22,23].

Different pharmacokinetics were observed in dogs by s.c. administration, where a stable dose dependent serum level was already reached within 30 min. This could be due to faster absorption compared with i.p. administration to rats. Surprisingly, it was possible to increase the plasma level further by repeated administration to 328 ng/mL (CI: 78 ng/mL, n = 6). This dose dependency is not easy to explain because BE was incompletely absorbed in all dose level groups. It is concluded that in spite of its low aqueous solubility TE, administered i.p. or s.c., gives rise to a plasma level of BE.

Apart from slight inflammation due to the undissolved particles, no toxicity was found in subchronic toxicity studies. This is in line with previously published data for single triterpenes. For example i.p. administered OA has a LD50 of 1,500 µg/mL in mice [14] and a single s.c. dose of 1,000 µg/mL caused no toxic effects in rats [13].

## Conclusions

In conclusion, triterpene extract from birch bark is safe, its BE bioavailable and in addition to known triterpene activities, TE provides high potential for further pharmaceutical and pharmacological research. The desired route of administration is dermal why the skin penetration should be determined. Based on these preliminary pharmacokinetic studies the pharmacokinetics of TE should be studied within the dermal route in detail.

## Experimental

### General

Pharmacokinetic and toxicological studies were performed according to ICH guidelines, the Japanese Guideline for Non-clinical Studies of Drugs (1995) and guidelines for toxicity studies of drugs (Japanese Ministry of Health and Welfare) at LPT Laboratory of Pharmacology and Toxicology GmbH & Co. KG, Hamburg, Germany. Plasma samples were obtained during several subchronic toxicological studies with TE in Beagle dogs (Stefano Morini S.A.S., San Polo d’Enza, Italy) and Sprague-Dawley rats (Charles River Deutschland, Sulzfeld, Germany). Animal tests were performed according to European laws and regulations. GC-MS was used for quantification of betulin (BE) within plasma samples.

### Plant material, extraction and characterization of triterpene extract

Outer bark of birch from various sources was identified as described [2]. A voucher specimen of each batch was deposited in the archive of Birken GmbH, Niefern-Öschelbronn, Germany. TE was obtained by accelerated solvent extraction with *n-*hexane at 1450 psi and 120 °C after drying the precipitate at 80 °C. TE extracted with *n*-heptane was extracted by a continuous procedure and provided by Birken GmbH, Niefern-Öschelbronn, Germany [2]. Its chemical characterisation was performed by GC-FID as recently published [2]. Aqueous TE solubility was measured by adding 200 µg/mL TE to boiling distilled water. After saturation at room temperature, the triterpenoid concentration was determined by GC-FID [16].

### Animal studies: Preliminary pharmacokinetics

Thirty six Sprague-Dawley rats were treated with TE for 4 weeks in 3 dose level groups (see Table 2). Blood sampling from the retrobulbar venous plexus was done at the times listed in Table 2. The plasma samples (approx. 0.5 mL EDTA plasma/animal/sampling time) were immediately frozen and stored at –20 °C. Dog blood was collected from the cephalic vein of the right or left forelimb. Blood was sampled on test day 1 (pre-dose) and day 28 (end of study) from all animals in each dose level group, at the time points shown in Table 2. Approx. 0.5 mL EDTA plasma was immediately frozen and stored at –20 °C.

**Table 2:** Dose schedules of TE for the determination of BE in plasma.

administration	animal	suspension medium	dosis schemata	blood sampling
intraperitoneal (i.p.)	sprague-dawley rats (3 female, 3 male) per dose level group	sesame oil	60, 180, 540 g/kg, vol. 10 mL/kg, for 28 days daily	day 1: 0.5, 1, 2, 4 h day 28: 0, 0.5, 1, 2, 4 h
subcutan (s.c.)	beagle dogs (3 female, 3 male) per dose level group	PEG 400 / 0.9 % NaCl	30, 100, 300 mg/kg, vol. 5 mL/kg, for 28 days daily	day 1: 0.5, 1, 2, 4 h day 28: 0, 0.5, 1, 2, 4 h

### Quantification of betulin within plasma samples (GC-MS)

Internal standard (0.5 µg/mL in methanol, 10 µL, ER Roth, Karlsruhe, Germany) was added to plasma sample (500 µL). 0.9 % NaCl solution (500 µL) was added. The mixed samples were transferred onto a C8-SPE extraction cartridge (Bond Elut C-8, 500 mg, 3 mL, Varian, Darmstadt, Germany) and washed with methanol/water (20/80, v/v, 2 mL). The sample was extracted from the C8-cartridge with methanol (3 mL) and evaporated to dryness. The residue was reconstituted in chloroform (200 µL) using a mixer and for 1 min in an ultrasonic bath. Afterwards *n-*hexane (1.8 mL) was added and this extract was transferred to a Si-SPE-cartridge (Bond Elut Si, 500 mg, 3 mL, Varian) and washed with *n-*hexane/ethyl acetate (90/10, v/v, 2 mL). The sample was extracted from the Si-cartridge with *n-*hexane/ethyl acetate (25/75, v/v, 3 mL) and evaporated to dryness. The residue was reconstituted in derivatization reagent (*N-*Methyl-*N-*trimethylsilylheptafluor(o)butyramide, 100 µL) with a mixer and for 1 min in an ultrasonic bath. An aliquot (1 µL) was separated by GC using a HP 6890 plus (Agilent Technologies, Germany) equipped with a ZB-35 column (Phenomenex, Germany, 30 m x 250 µm x 0.25 µm). The carrier gas was helium (quality 5.0) at a constant flow of 1.2 mL/min. Split injection (20:1) was employed at 250 °C and 14.78 psi. Separation was obtained by the oven program: 130°C (1 min constant); a ramp of 20 °C/min to 300 °C (17.5 min constant).

Detection took place using mass selective detection (HP 5973, Agilent Technologies) in the single ion monitoring mode at m/z 393.3, 483.3, 571.4 and 586.4. The transfer line was heated to 300 °C, the MS-Quad to 150 °C and the MS-source to 230 °C. The multiplier voltage was set to 1450 V, the electron impact ionisation to 70 mV and the dwell was set to 100. Standard calibration with BE and ER added to plasma (BE: between 10 and 4992 ng/mL; ER: 50 ng/mL) was used for peak identification and quantification. See key data on validation in Table 3.

**Table 3:** Validation.

validation parameter	quantification of BE in plasma
specificity	as required
linearity	r = 1.000
accuracy	approx. ± 95 % (mean)
intra-day- and, inter-day precision	approx. ± 95 % respectively
stability	no apparent degradation within 168 h of storage, 24 h of storage in the derivatization reagent
recovery	BE: 94.9 ± 4.2 %
	ER: 71.3 ± 2.8 %
sensitivity, limit of quantification (LOQ)	0.05 µg/mL

It was possible to use ER as an internal standard even though it is a component of TE (approximately 1 %), because at maximum observed BE serum concentrations of 1.20 µg/mL the ER concentration provided by TE would be 0.012 µg/mL which is below the limit of quantification (0.05 µg/mL).

### Subchronic toxicity studies

For the subchronic toxicity studies the same scheme was used as described in Table 2 but the number of rats was 10 per sex and dose level groups and a vehicle control dose level group was added within each study. During the course of the experiment the animals were observed for clinical symptoms, mortality, body weight, food and drinking water consumption. Haematological parameters were determined by an analyzer (Sysmex KX-21, Sysmex Deutschland GmbH, Norderstedt, Germany). Differential blood count was performed by quick staining [24]. For the pathological and histopathological observations animals were sacrificed and dissected on test day 29 (or 30) 24 hours after the last administration.

### Statistics

Statistical calculations were carried out with Excel 2000 and Validat 2000 (Headwork-Consulting, ICD; Frechen, Germany). Results are expressed as mean ± standard deviation (SD) or confidence interval (CI, α = 0.05) of n independent experiments. Statistical significance is established at values of p ≤ 0.05 marked by a single asterisk whereas high significance (p ≤ 0.01) is shown by two asterisks. The multiple t-test based on Dunnett test (p ≤ 0.01) was used for calculations of body weight, food consumption, clinical biochemistry and relative organ weights.
